# Analysis of Anti-desmoglein 1 Autoantibodies in 68 Healthy Mother/Neonate Pairs from a Highly Endemic Region of Fogo Selvagem in Brazil

**DOI:** 10.4172/2155-9554.1000209

**Published:** 2014-02-24

**Authors:** Julio Hilario-Vargas, Irineu B Vitorio, Christopher Stamey, Donna A Culton, Phillip Prisayanh, Evandro A Rivitti, Valeria Aoki, Gunter Hans Filho, Vandir dos Santos, Bahjat Qaqish, Luis A Diaz

**Affiliations:** 1 Departments of Physiology, National University of Trujillo School of Medicine, Peru; 2 Departamento de Obstetrics, Universidade Federal de Mato Grosso do Sul, Brazil; 3 Department of Dermatology, University of North Carolina at Chapel Hill, Chapel Hill, NC, USA; 4 Departamento de Dermatologia, Universidade de Sao Paulo, Brazil; 5 Departamento de Dermatologia, Universidade Federal de Mato Grosso do Sul, Brazil; 6 Department of Biostatistics, University of North Carolina at Chapel Hill, NC, USA

**Keywords:** Fogo Selvagem, Pemphigus Foliaceus, Neonatal Pemphigus, Neonatal Autoimmunity, Autoimmune Bullous Diseases, Pemphigus Vulgaris

## Abstract

**Objectives:**

Fogo Selvagem (FS) in Limao Verde (LV), Brazil shows clinical and histological features of pemphigus foliaceus (PF) and shares pathogenic IgG4 anti-desmoglein 1 (Dsg1) autoantibodies. Previously, our group reported that mothers with active FS deliver babies with normal skin and low/negative titers of IgG4 autoantibodies by indirect immunofluorescence. It was postulated that maternal pathogenic IgG4 autoantibodies do not cross the placenta due to differential receptor mediated transplacental passage of IgG subclasses. It was also thought that placental Dsg1 may immunoadsorb pathogenic autoantibodies from the mother; hence pathogenic IgG4 autoantibodies do not reach the baby.

In this study we use a Dsg1-specific ELISA to test anti-Dsg1 autoantibodies of the IgM, IgG and the IgG subclasses in the sera of 68 pairs of normal mothers and their neonates living in a highly endemic area of FS. Determination of these baseline anti-Dsg1 autoantibodies will allow us to follow and predict in this and other cohorts the appearance of preclinical serological markers of FS.

**Methods:**

The sera of mothers and neonates living in the endemic region were tested by ELISA for IgM, IgG and IgG subclasses using recombinant Dsg1 and anti-IgG subclass-specific monoclonal antibodies.

**Results:**

The index values of anti-Dsg1 IgG1, IgG2 and IgG3 are similar in mothers and neonates (all p>0.18), while the index values of IgM, total IgG and IgG4 are higher in mothers (all p<0.001).

**Conclusions:**

Narrowing the IgM, IgG and IgG subclasses of mothers and neonates to autoantibodies against Dsg1, we found, as expected, that IgM remains only in maternal circulation. In three mothers and two neonates we detected IgG4 anti-Dsg1 autoantibodies above the normal range. The remaining IgG subclasses show low values. The results of the neonatal sera will serve as a baseline for ongoing seroepidemiological studies of children and adults in the endemic regions of FS.

## Introduction and Review of the Literature

Fogo Selvagem (FS) in Limao Verde (LV), Brazil shows clinical and histological features of pemphigus foliaceus (PF) and shares pathogenic IgG4 anti-desmoglein 1 (Dsg1) autoantibodies [1-3]. Additionally, many healthy inhabitants from LV possess IgG and IgM anti-Dsg1 autoantibodies [4-6]. The factors that trigger the transition from non-pathogenic IgG1, IgG2 and IgG3 responses to pathogenic IgG4 autoantibodies in these individuals remain unknown; however, from the large pool of normal individuals living in LV, only a small fraction develop FS suggesting that disease occurs in those individuals that are genetically predisposed [7]. Compelling epidemiological and serological evidence suggests that FS is precipitated by exposure to bites of hematophagous insect, endemic to the same regions of FS [8].

Previously, our group examined the skin and the sera of 19 neonates of mothers with active FS clinically and by direct and indirect IF techniques [9]. All neonates were born with normal appearing skin, whereas the mothers showed classic skin lesions of FS. The sera of all neonates were negative or showed substantially weaker titers of IgG4 autoantibodies compared to their mothers. It was hypothesized that absence of skin disease in the newborns was due to low transfer of IgG4 autoantibodies through the placenta and conceivably by an “immunosorbent” effect of the placenta that is known to contain desmosomes and desmogleins [10-12]. Further supporting this theory, Oyama et al. have shown that placental tissue binds anti-Dsg 1 antibodies in patient serum [13].

Healthy inhabitants settled in endemic regions of FS possess IgG and IgM autoantibodies in childhood suggesting that exposure to an environmental antigen(s) occurs early in life; after an incubation period that last from 1 to 12 years susceptible individuals will develop FS [4]. It is of utmost importance to establish the serological markers of anti-desmoglein autoantibodies at time zero, i.e. at birth. Thus, prospective evaluation of cohorts in Limao Verde, as shown in our studies would indicate that the immunization process in inhabitants of this endemic settlement begins in the first few years of life [4]. In this study we evaluate the IgM and IgG anti-Dsg1 autoantibody responses in 68 normal mother/newborn pairs seen at Aquidauana Hospital in Brazil, located 30 kilometers southeast of LV.

## Materials and Methods

Peripheral blood from mothers and umbilical blood from neonates was collected during delivery by the obstetrician (IBV). The sera were transported and stored at −20°C at the University of North Carolina Dermatology Research Laboratories until tested. The Ethics Committees and the Institutional Review Boards of the Universities of North Carolina and Sao Paulo and the National University of Trujillo approved these studies.

The sera of mothers and neonates were tested by indirect IF and by ELISA for IgM, IgG and IgG subclasses using recombinant Dsg1 (rDsg1) and anti-IgG subclass-specific monoclonal antibodies with results expressed as index value units [2,4,5]. Previously published cutpoints with unique sensitivity (sen) and specificity (spe) were used to define positive sera; IgM: 50 (sen: 50%, spe: 96%), IgG: 11 (sen:87%, spe: 91%), IgG1: 11 (sen:72, spe:80), IgG2: 4.5 (sen: 49, spe: 54), IgG3: 5 (sen: 62, spe: 65), and IgG4: 6.4 (sen: 92, spe: 97) [2,4,5].

## Results

**Figure 1:**
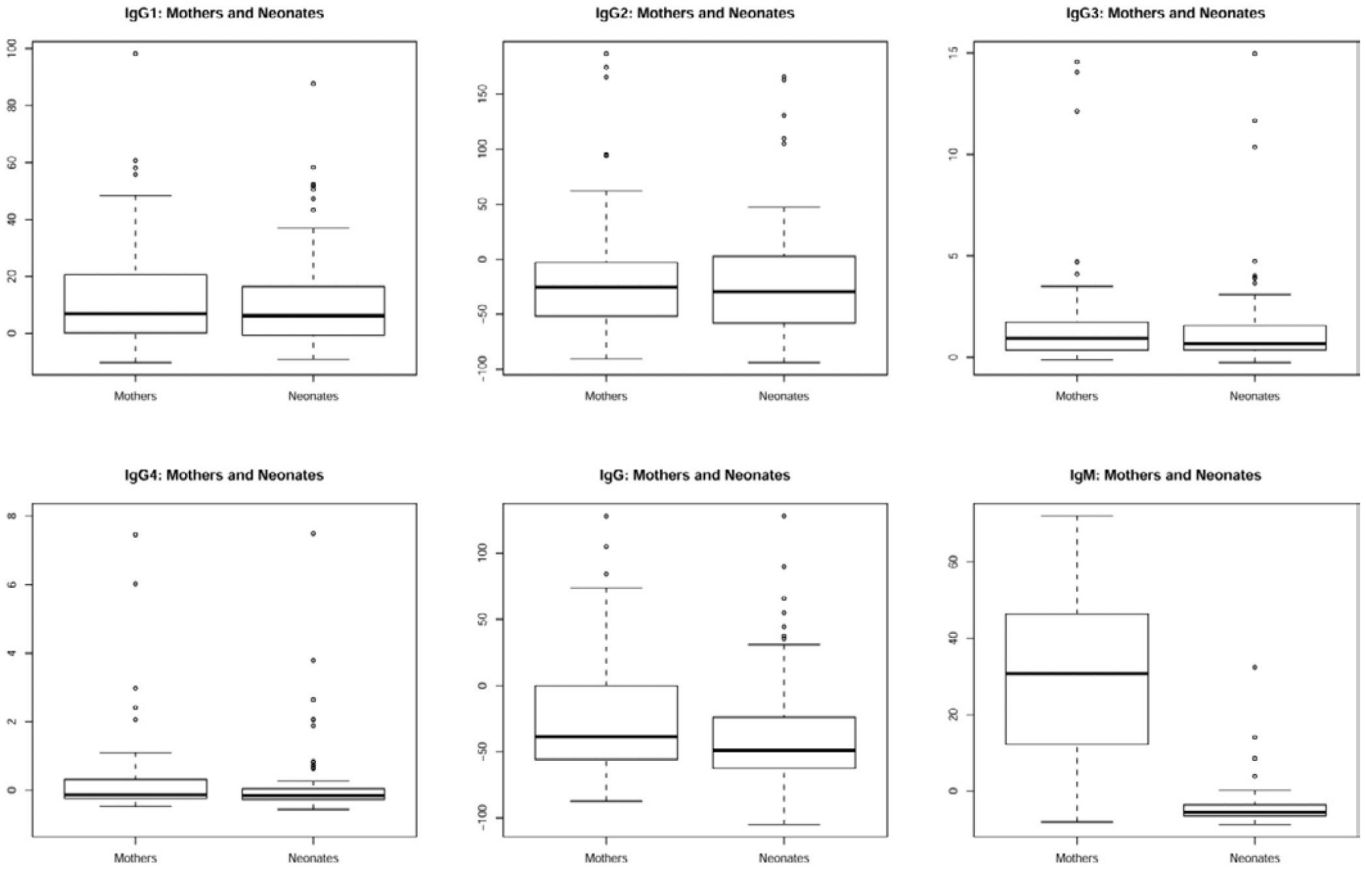
Distribution of Index Values for IgG subclasses, total IgG and IgM anti-Dsg1 autoantibodies in mothers and neonates.

The sera from the 68 pairs are negative by indirect IF against human skin substrate (data not shown). The Dsg1 ELISA results for individual autoantibodies classes/subclasses are shown as boxplots in Figure 1 for mothers (left) and neonates (right). The index values of IgG1, IgG2 and IgG3 are similar in mothers and neonates (all p>0.18), while the index values of IgM, total IgG and IgG4 are higher in mothers (all p<0.001). The percentage of cases in which the mother’s autoantibody level exceeds their neonate’s is 60.2% for IgG4, 68% for total IgG and 87% for IgM. Using the cutpoints, we find only two neonates from three of the mothers who were positive for IgG4 anti-Dsg1 autoantibodies (>6.4) showed positive index values (Table 1).

## Discussion

In this investigation, we demonstrate that IgG and IgG-subclass anti-Dsg1 autoantibodies are present in the sera of normal mothers and their newborns living in a highly endemic region of FS en Brazil. The differences between mothers and neonates, and the correlation pattern observed, suggest that all IgG anti-Dsg1 autoantibodies cross the placental barrier in low levels, except IgM anti-Dsg1 autoantibodies which is undetectable in neonates. These data also indicate that while similar levels of anti-Dsg1 IgG1-3 autoantibodies are present in mothers and neonates, IgG4 autoantibodies were detected only in 3 mothers (4%) and 2 neonates (3%). These individuals are under surveillance, evaluating for a sustained IgG4 anti-Dsg1 response that may predict FS in ~50% of the cases [2]. It may be assumed that all IgG subclasses, including those bearing anti-Dsg1 specificity, detected in the newborn sera are derived from their respective mothers as the neonatal immune system is naïve, immature and not yet producing appreciable amounts of IgG.

A short-lived skin disease of newborns has been described in babies from mothers with pemphigus vulgaris (PV), herpes gestationis, and occasionally in PF [14-17]. However, disease has not been reported in babies from mothers with active FS [9]. A possible explanation for this observation is that transplacental passage of IgG subclasses is regulated by an FcRn receptor mechanism which allows crossing of IgG subclasses at different rates (IgG1>IgG4>IgG3>IgG2) [10,18]. Alternatively, it is hypothesized that IgG4 anti-Dsg1 autoantibodies from the mother are retained by the placenta, which would act like an “immunoadsorbent” for pathogenic autoantibodies given that the placenta expresses desmosomes and desmogleins [11,12]. However, neonatal disease has been seen in PF indicating that there is sufficient passage of anti-Dsg1 IgG4 antibodies in these cases and presence of the target antigen in neonatal skin necessary to produce disease [16,17]. Differences in the intrinsic physical properties of the autoantibodies in FS such as affinity, avidity, or conformation, could also lead to different pathogenic potential for the IgG4 subclass.

Based on our studies, exposure to the putative environmental antigen(s), i.e. salivary proteins from insects likely occurs in early childhood and leads to the generation of nonpathogenic IgG, IgG1, IgG2 and IgG3 and IgM anti-Dsg1 autoantibodies [4]. The majority of these individuals may remain disease-free and will never develop clinical disease; however, in a minority of genetically-predisposed individuals the immune response may lead to the generation of pathogenic IgG4 anti-Dsg1 autoantibodies. The present study demonstrates that the IgM and IgG anti-Dsg1 autoimmune response in neonates born in an endemic area of FS is negative or at low levels. Consequently, we can assume that FS occurs in predisposed hosts exposed to an environmental antigen after the first few years of life.

## Figures and Tables

**Table 1:** Number and percentages of individual autoantibody systems above the cutpoints (positive sera) as determined by ROCs from index values of anti-Dsg1.

Autoantibodies	Mothers (n=68)	Neonates (n=68)
IgM	12 (16%)	0 (0%)
IgG	13 (17%)	11 (14%)
IgG1	26 (38%)	23 (34%)
IgG2	13 (17%)	17 (25%)
IgG3	8 (12%)	8 (12%)
IgG4	3 (4%)	2 (3%)
